# Spirit Interim Analysis: A Multicenter Prospective Observational Study of Outpatients with CKD and Decreased eGFR to Assess Therapeutic Algorithms, Disease Management and Quality of Life in Greece

**DOI:** 10.3390/jcm14062079

**Published:** 2025-03-18

**Authors:** Dimitrios Petras, Smaragdi Marinaki, Stylianos Panagoutsos, Ioannis Stefanidis, Kostantinos Stylianou, Evangelia Ntounousi, Sofia Lionaki, Ioannis Tzanakis, Ioannis Griveas, Dimitrios Xidakis, Eleni Theodoropoulou, Dimitris Gourlis, Argyris Andreadellis, Dimitrios Goumenos, Vassilios Liakopoulos

**Affiliations:** 1 Nephrology Department, Hippokration General Hospital, 11527 Athens, Greece; 2 Department of Nephrology, Laiko General Hospital, National and Kapodistrian University School of Medicine, 11527 Athens, Greece; smaragdimarinaki@yahoo.com; 3 Department of Nephrology, University Hospital of Alexandroupolis, 68100 Alexandroupoli, Greece; spanagou@med.duth.gr; 4 Department of Nephrology, University Hospital of Larissa, 41334 Larissa, Greece; stefanid@med.uth.gr; 5 Department of Nephrology, University Hospital of Heraklion, 71500 Heraklion, Greece; kstylianu@gmail.com; 6 Department of Nephrology, School of Medicine, University of Ioannina, 45110 Ioannina, Greece; edounous@uoi.gr; 7 Department of Nephrology, Attikon University Hospital, National and Kapodistrian University of Athens, School of Medicine, 12462 Athens, Greece; sofia.lionaki@gmail.com; 8 Department of Nephrology, General Hospital of Chania, 73300 Chania, Greece; ioatzan@gmail.com; 9 Nephrology Department, Army Share Fund Hospital of Athens, 417 NIMTS, 11521 Athens, Greece; giannisgriv@hotmail.com; 10 Department of Nephrology, Venizelio General Hospital of Heraklion, 71409 Heraklion, Greece; dxydakis@gmail.com; 11 Department of Nephrology, Tzaneio General Hospital, 18536 Piraeus, Greece; htheodoropoulou@yahoo.gr; 12 Medical Affairs Department, AstraZeneca, 15123 Athens, Greece; dimitris.gourlis@astrazeneca.com (D.G.); argyris.andreadellis@astrazeneca.com (A.A.); 13 Department of Nephrology, University Hospital of Patras, 26504 Patras, Greece; dgoumenos@upatras.gr; 14 Department of Nephrology, AHEPA Hospital, Aristotle University of Thessaloniki, 54636 Thessaloniki, Greece; liakopul@otenet.gr

**Keywords:** chronic kidney disease, disease management, observational prospective study, treatment patterns, quality of life

## Abstract

**Background:** Chronic Kidney Disease (CKD) affects 8–16% of the population worldwide and is characterized by an estimated Glomerular Filtration Rate (eGFR) of less than 60 mL/min/1.73 m^2^ for more than 3 months. The main purpose of the study is to record the treatment algorithms and disease management of patients presenting for the first time to hospital-based nephrologists with a reduced eGFR and CKD diagnosis, under real-world clinical practice in Greece. **Methods:** This is the 6-month interim analysis of an ongoing, multicenter, observational, prospective, national study, which included 178 patients, with an eGFR between <60 and 15 mL/min/1.73 m^2^, presenting for the first time to nephrologists at 15 public hospital units. **Results:** The median age of the patients was 71 years old, with 39.6% of them categorized as CKD stage G3b. Of these patients, 71.6% and 33.7% suffered from arterial hypertension and type 2 diabetes mellitus, respectively; 78.7% of patients received antihypertensive and 38.5% antidiabetic medications. Calcium channel blocker usage increased with disease progression (from 52.2% at G3a, to 67.9% and 67.6% at G3b and G4, respectively), while that of angiotensin II receptor antagonists decreased (from 78.3% at G3a, to 41.5% and 17.6% at G3b and G4, respectively). A decrease in metformin usage and an increase in Dipeptidyl peptidase-4 inhibitor (DPP4i) usage was also observed upon disease progression. Furthermore, 18.5%, 32.0% and 7.7% of patients received Sodium-glucose cotransporter-2 inhibitors (SGLT2i) at the G3a, G3b and G4 stages, respectively. **Conclusions**: The interim analysis results contributed to the collection of real-world data for the therapeutic patterns and the management of CKD in Greece.

## 1. Introduction

Chronic Kidney Disease (CKD) affects 8–16% of the population worldwide [1], amounting to >800 million individuals [2], and is defined as the presence of kidney damage for more than 3 months, reflected by an estimated Glomerular Filtration Rate (eGFR) of less than 60 mL/min/1.73 m^2^. In Greece, CKD is among the top 10 causes of death in the country [2], with an estimated over 1 million patients affected across all stages (1–5) [3]. The course of the disease progresses from glomerular hyperfiltration to progressive albuminuria, a declining GFR, and, ultimately, End-Stage Renal Disease (ESRD) [4]. The major risk factor for CKD is type 2 diabetes mellitus (T2DM), which causes microvascular and macrovascular damage to several organs and increases the risk of developing CKD by 2.6-fold [5].

A diagnosis of CKD is largely based on a reduced eGFR by abnormal findings on routine blood or urine tests (elevated serum urea and/or creatinine or albuminuria). An eGFR of less than 60 mL/min/1.73 m^2^ is considered abnormal for all adults; however, when co-presenting with albuminuria, urine sediment abnormalities or kidney imaging or biopsy findings, an eGFR of less than 90 mL/min/1.73 m^2^ is indicative of impaired kidney function [6].

KDIGO 2012 guidelines [7] for the general management of CKD for diabetic and non-diabetic CKD patients recommend to aim for lowering blood pressure, using angiotensin receptor blockers (ARBs) and angiotensin-converting enzyme inhibitors (ACE-I). Moreover, the KDIGO 2022 Clinical Practice Guidelines for Diabetes Management in CKD recommend the use of SGLT2 inhibitors for most patients with T2DM, CKD and an eGFR ≥ 20 mL/min per 1.73 m^2^ [8,9]. The current KDIGO guidelines of 2024 introduce a more general recommendation of treating CKD with an SGLT2i, if the eGFR ≥ 20 mL/min per 1.73 m^2^, with a Urine Albumin Creatinine Ratio (UACR) ≥ 200 mg/g (≥20 mg/mmol) or heart failure, irrespective of level of albuminuria [8]. The same guidelines also suggest SGLT2i in adults with an eGFR ≥ 20 to 45 mL/min/1.73 m^2^, with a UACR < 200 mg/g. As CKD is associated with a relatively high morbidity and mortality rate, treatment patterns and the evidence as to how CKD management affects patient’s quality of life are of crucial importance. In Greece, CKD constitutes an increasing burden on the National Healthcare System, as it confers one of the highest ESRD incidence rates among developed countries [10].

Several complications have been previously associated with progressive CKD, leading to high mortality, morbidity and a poor quality of life [11]. Some of these conditions can be easily characterized and quantified (such as cardiovascular disease, anemia, hypertension, mineral bone disorder, acid-base abnormalities, volume overload, electrolytes) and, therefore, managed. Others, such as sexual dysfunction, fatigue, anorexia, pruritus and nausea, are less well defined, with a more controversial etiology, and frequently present as complex symptoms related to more advanced CKD [11].

Hyperkalemia is a rare condition in the general population but a common complication in CKD [12], with patients suffering from diabetes and heart failure and those receiving Renin–Angiotensin–Aldosterone System inhibitors (RAASis) being at a higher risk of developing it [13,14,15]. In a survey among cardiologists and nephrologists, hyperkalemia was reported in 42% of chronic HF (heart failure) patients and 50% of CKD patients, with CKD patients showing a trend toward more severe hyperkalemia. Chronic hyperkalemia was present in the majority of hyperkalemic patients (60%) [16]. Patients with mild hyperkalemia usually present without signs or symptoms, but at higher potassium levels, hyperkalemia can be associated with life-threatening cardiac dysrhythmias, muscle weakness or paralysis. In contrast, patients with chronic hyperkalemia such as those undergoing dialysis may remain asymptomatic, despite marked elevations in potassium [17].

## 2. Materials and Methods

### 2.1. Study Design and Objectives

The SPIRIT study is an ongoing multicenter, observational, prospective, national study collecting information on routine clinical practice and treatment patterns for managing a decreased eGFR and CKD in a real-world setting in Greece. This study enrolled the first patient at the General Hospital of Heraklion “Venizeleio-Pananeio” and was approved by the Scientific Council of the General Hospital of Heraklion “Venizeleio-Pananeio”, Greece (Ref. number 28/17-11-2022). The study was conducted in compliance with local and national regulations, in accordance with the Declaration of Helsinki, ICH GCPs, GPP and the applicable legislation on non-interventional studies and/or observational studies. All patients provided written informed consent and were made fully aware that they could withdraw from the study at any time without any consequences for future care. Table 1 shows the primary and secondary objectives of the study.

The study is registered on the electronic Registry of Non-Interventional Studies (RNIS) and posted on the website of the Hellenic Association of Pharmaceutical Companies (SFEE) (https://www.dilon.sfee.gr/studiesp_d.php?meleti_id=D1843R00342 (accessed on 11 March 2025)).

### 2.2. Study Population and Eligibility Criteria

During a 9-month recruitment period, the study enrolled 178 patients with an eGFR between 15 and 59 mL/min/1.73 m^2^ who presented for the first time to 15 public hospital-based Nephrology outpatient clinics throughout the country, being either primary nephrological cases or referrals by other specialties. To avoid introducing patient selection bias, physicians consecutively enrolled the first 10 patients attending their hospital/clinic that met the eligibility criteria. Inclusion and exclusion criteria are illustrated in Table 2 The interim analysis was performed after 50% of the patients were enrolled (data cut-off date), and it analyzed data from 169 out of the 178 patients, as 4 patients were not eligible for the study and 5 patients had missing data for the CKD-EPI eGFR. The total study population is planned to include 305 patients.

### 2.3. Data Source

Sites and investigators were selected to represent the management of patients with a decreased eGFR and CKD at a national level, covering major geographic areas. The final selection of the participating sites was based on a documented feasibility evaluation process that assessed physicians’ qualifications and previous participation and experience in similar clinical studies. Data were obtained prospectively during the study visits as performed per common clinical practice or through PRO questionnaires. Printed copies of the PROs were completed by the patients at the beginning of the visit before the patient’s status was discussed with the physician. Data regarding the patient’s medical history were collected retrospectively from patient medical charts/records. Physicians followed eligible patients and recorded their management according to their usual clinical practice. Only medical records from routine clinical practice as provided by the physicians were used as source documentation for this study. The data were subsequently entered into the electronic Case Report Form (eCRF), ensuring that the collected information accurately reflected real-world clinical practice. A past medical history recording that included T2DM, arterial hypertension, coronary artery disease, depression, dyslipidemia, hypothyroidism, hyperuricemia, atrial fibrillation, heart failure and osteoporosis provided a comprehensive picture of the comorbidity landscape within the study population (prior and/or after study enrolment). Additionally, the hematological parameters of the patients were also examined across all CKD stages.

### 2.4. Statistical Analysis

The present study was descriptive; therefore, no formal hypothesis was tested. Descriptive statistics were used for the presentation of the data. For continuous variables, the mean, standard deviation and range or median, 25th–75th percentiles and range were calculated, depending on the outcome of testing for normal distribution. The frequencies and percentages were presented regarding categorical data. Missing data (number and percentage) were included in the tables. However, missing data were not included in the denominator for calculating percentages of the categorical data. All analyses were performed by a designated contract research organization (CRO) with the statistical package of SAS^®^ (Version 9.1).

## 3. Results

### 3.1. Patient Characteristics

A total of 169 patients were included in the study’s interim analysis. Patients were categorized as G3a (37.9%), G3b (39.6%) and G4 (22.5%) based on eGFR calculations and had a median age of 71 years. Gender distribution revealed that males were the predominant gender in the total population, with percentages ranging from 47.4% in G4 to 81.3% in G3a. Baseline characteristics are shown in Table 3.

Each CKD stage was examined in terms of smoking behavior, alcohol consumption and dietary patterns with low salt intake. Most patients had never smoked (52.7%), and the median packs per day was slightly higher for the smokers of stage G4 (1.3 vs 1.0 for G3a and G3b). Regarding alcohol intake, most patients of all three CKD stages reported consuming alcohol in moderation, defined as “<1 unit per day” (88 (52.1%)), followed by those that abstained entirely from alcohol consumption (71 (42.0%)). In addition, patients consciously chose a low salt intake diet as an integral component of their daily regimen ((G3a (57.8%), G3b (50.8%), G4 (65.8%)).

### 3.2. CKD History and Comorbidities

Urine microalbumin, urine creatinine and UACR measurements were conducted at most six months before the baseline, revealing a median (25, 75 percentile) UACR level of 158.6 (31.1, 809.8) mg/g. T2DM and arterial hypertension were reported to be the leading causes of reduced eGFR for 27.1% and 45.9% of patients, respectively.

Notable comorbidities included arterial hypertension and diabetes mellitus type 2, which both affected a significant proportion of patients. Other comorbidities, such as coronary artery disease, depression, dyslipidemia, hypothyroidism, hyperuricemia, atrial fibrillation, heart failure, and osteoporosis provided a comprehensive picture of the comorbidity landscape within the study population and are presented in Table 3.

### 3.3. Laboratory Assessments and Physician’s Findings

All CKD patients were examined regarding various hematological parameters as presented in Table 3. In terms of hemoglobin levels, there was a noticeable decline as CKD progressed, with the mean (SD) hemoglobin decreasing from 13.1 (1.73) g/dL in G3a to 11.5 (1.49) g/dL in G4. Hematocrit levels demonstrated a similar pattern, where G3a had a higher mean compared to G3b and G4. The red blood cell count (RBC) and white blood cell count (WBC) levels followed a similar trend, with a decrease in mean RBC and mean WBC as CKD advanced. The mean corpuscular volume (MCV) and platelets remained relatively stable across the CKD stages and within the expected range, with minimal variations.

The serum biochemistry test results in patients across different CKD stages revealed notable variations. Albumin demonstrated a pronounced decline in advanced CKD (G4), with a median (25, 75 percentile) of 4.0 (3.8, 4.3) g/dL, signaling hypoalbuminemia, while urea and Parathyroid hormone (PTH) levels rose as CKD progressed, with G4 patients having a median (25, 75 percentile) of 109.0 (90.0, 139.0) mg/dL and 117.0 (85.0, 194.0) pg/dL, respectively. Glycated hemoglobin (HbA1C), Total Cholesterol and Triglyceride levels showed variability between stages, while glucose, high-density lipoprotein (HDL) levels and some other parameters (potassium, sodium, calcium, phosphorus) remained relatively stable.

### 3.4. Health Related Patient Reported Quality of Life (QoL)

The EQ-5D-5L (VAS scale) and KDQOL-SF questionnaires (Health Rate scale) were completed by the majority of patients across all three CKD stages, with 98.8% and 99.4% of the total sample participating, respectively. The mean scores for the VAS scale and the Health Rate scale were comparable between different stages, with G3a scoring slightly higher for both questionnaires: 74.6 and 7.0, respectively, as shown in Table 4.

### 3.5. Treatment Patterns Across CKD Stages

The treatment patterns of patients across different CKD stages included antidiabetic medications that were administered to 65 patients (38.5%). DDP4 inhibitors in 25 patients (38.5%) and metformin in 21 patients (32.3%) were the most administered drugs. SGLT2 inhibitors are included in the table as an antidiabetic medication, since it is not specified by the protocol for which indication they are administered. Therefore, it is possible for SGLT2 inhibitors to be administered for CKD treatment as well as their antidiabetic effect. Similarly, RAAS inhibitors are included in the table as antihypertensive medication, but it is possible for them to be administered as a CKD treatment. Antihypertensive medications were administered to 133 patients (78.7%), with their usage increasing according to the CKD stage, i.e., from 46 patients (71.9%) out of 64 patients in G3a, to 34 patients (89.5%) out of 38 patients in G4. The specific types of antihypertensive medications varied within each CKD stage. Calcium channel blockers in 83 patients (62.4%) and angiotensin II receptor antagonists in 64 patients (48.1%) were the most administered drugs. The treatment patterns followed by all participants are presented in Table 5.

## 4. Discussion

The SPIRIT study is an ongoing multicenter, prospective, observational study of outpatients with CKD and a decreased eGFR to assess treatment patterns, disease management and quality of life in Greece. The interim analysis was performed when 50% of the patients were enrolled, and it analyzed baseline characteristics data.

Regarding the management of patients with CKD, antidiabetic medications, including DDP4 inhibitors and metformin, were prescribed. Notably, metformin was more commonly administered in the earlier stages; however, its usage decreased in advanced CKD stages, aligning with the KDIGO 2022 guidelines for CKD [9] to mitigate the risk of lactic acidosis. Other antidiabetic medications, mostly used in the G3a and G3b stages rather than in G4, included SGLT2 inhibitors and GLP-1 analogues, highlighting the diversity in antidiabetic treatment choices among CKD patients. The observed reduction in the use of SGLT2i across CKD stages may be partially attributed to the KDIGO guidelines’ recommendations, which advocate for initiating SGLT2i treatment in patients with type 2 diabetes and CKD when the eGFR ≥ 20 mL/min per 1.73 m^2^; therefore, patients with an 15 < eGFR < 20 mL/min per 1.73 m^2^ are excluded [8]. These findings are also in agreement with the results from the KDICO descriptive study in Colombia, which examined the treatment patterns of antidiabetic and kidney-protective therapies among patients with type 2 diabetes mellitus and CKD. In this study, metformin, either as a monotherapy or in combination with other antidiabetic agents, was the most commonly prescribed treatment for TDM2 among CKD patients, followed by Dipeptidyl peptidase-4 (DPP4) inhibitors, some type of insulins (particularly long-acting and short-acting analogues) and sulfonylureas. In the group of drugs with a positive impact on kidney function, the use of SGLT2 inhibitors and GLP-1 analogues was notable [18]. Another retrospective cohort study using a national-level claims dataset (MarketScan Commercial and Medicare Supplemental databases) and electronic health records [IBM MarketScan Explorys Claims–Electronic Medical Record (EMR)] demonstrated similar real-world evidence regarding general treatment practices of diabetic kidney disease in the United States. In particular, around 78% to 80% of patients across all eGFR levels were treated with glucose-lowering drugs, with metformin, sulfonylurea, DPP4 inhibitors and insulin being the most used agents (>10% across all eGFR levels). On the other hand, thiazolidinedione, glucagon-like peptide-1 receptor agonists (GLP-1 RAs), SGLT2 inhibitors or meglitinide were used by less than 10% of patients [19].

Antihypertensive medications were prevalent, administered to 133 patients (78.7%), with their usage increasing with CKD progression, from 46 patients (71.9%) in G3a to 34 patients (89.5%) in G4. The specific types of antihypertensive medications varied across CKD stages, with calcium channel blockers and angiotensin II receptor antagonists being the most commonly administered drugs. Other antihypertensive medications included adrenergic receptor antagonists, diuretics, and ACE inhibitors. These treatment patterns are in line with the recommendations of the KDIGO 2024 guidelines for CKD [8] and KDIGO 2021 guidelines for the management of blood pressure in CKD [20] that indicate the use of ACE inhibitors or angiotensin II receptor blockers as a first-line therapy for blood pressure control when albuminuria is present. Alternatively, dihydropyridine calcium channel blockers or diuretics are also considered as a therapeutic approach. Interestingly, a multicenter prospective study involving 39 hospitals in China (C-STRIDE) demonstrated that similar antihypertensive treatment patterns were administered to CKD patients. In this study, renin–angiotensin system inhibitors (RASIs), including angiotensin-converting enzyme inhibitors (ACEIs) and angiotensin receptor blockers (ARBs), were the most prescribed antihypertensive drugs (71.2%) among those 2213 C-STRIDE participants, followed by calcium channel blockers (67.9%), β-blockers (33.1%), diuretics (10.2%) and α-blockers (3.3%) [21]. Importantly, an advanced age, type 2 diabetes mellitus and hypertension are the primary risk factors for both CKD progression and the eventual need for hemodialysis. These findings highlight the critical role of conducting adequate metabolic control, integrating lifestyle changes and the use of antidiabetic medications that also have a positive impact on the progression of kidney damage alongside cardiovascular treatment that also protects kidney function.

The EQ-5D-5L questionnaire (VAS scale) and the KDQOL-SF questionnaire (Health Rate scale) were completed by most patients across all CKD stages, providing valuable insights into the self-assessed health and quality of life of patients with a high level of participation and engagement. The mean scores for the VAS scale and the Health Rate scale were comparable between different stages. These findings are in accordance with the results of a prospective observational study that revealed that the EQ-5D index score decreased (worsened) with more advanced CKD stages, but no significant difference was observed between CKD stages and the EQ VAS [22]. Another hospital-based cross-sectional study revealed that among the domains that constitute the KDQO-SF-36 physical and mental component summaries, lower scores in the SF-36 domains were associated with higher CKD stages. In addition, an advanced stage of CKD was found to be a predictor of worse physical functioning, social functioning, vitality and bodily pain [23].

Regarding patient characteristics, the predominant gender in each CKD stage was males, consistent with another study in Greece [24]; both reflect the prevalence of the disease. Lifestyle habits have also been examined regarding their contribution as risk factors for the occurrence of the disease. Physical activity is considered a controversial aspect in CKD by contrasting studies because of the risk of increased proteinuria and renal function impairment [25,26,27,28]. In addition, smoking had been associated with a worse progression in patients with established CKD by a Korean prospective cohort study. The study reported a higher risk of CKD progression with higher pack-years, showing a dose–response relationship between smoking and CKD. Similarly, current smokers have a greater risk of CKD progression than people who stopped smoking or have never smoked [29,30]. In terms of alcohol intake, the studies reported that persistent moderate alcohol drinking is not related to the progression of CKD compared to not drinking. However, binge alcohol drinking increases the risk of CKD progression [31,32].

Type 2 diabetes mellitus and arterial hypertension were the two main comorbidities affecting 46 (27.1%) and 78 patients (45.9%), respectively. These results are consistent with findings from certain meta-analyses, which identify diabetes mellitus and hypertension as the most common complications, risk factors and the leading causes of CKD in high-, middle- and low-income countries in the developing world [33,34,35,36].

The limitations of this interim analysis should be taken into consideration. The main limitation is related to the fact that this is a multicenter and observational study, carrying the possibility of patient selection bias and missing data. Moreover, as the study included real-world data from approximately 15–20 hospital sites and different laboratories within Greece, regional differences in healthcare practices, resource availability and patient demographics might have influenced the observed treatment patterns. Therefore, a valuable direction for future research could be to assess consistency and variability across healthcare centers and patient subpopulations, to provide deeper insights into national and local clinical practice trends.

## 5. Conclusions

In conclusion, the findings of the present interim analysis are in accordance with the current literature regarding the severity and staging of CKD, the patients’ underlying comorbidities and routine treatment approaches. These data and the subsequent overall study conclusions could possibly provide a direction in terms of risk factor management, screening and therapeutic strategies in CKD patients, further improving their clinical status and outcome.

## Figures and Tables

**Table 1:** Primary and secondary objectives.

**Primary objective**
- To describe treatment patterns and disease management following the presentation of patients with a CKD diagnosis at a Nephrology outpatient clinic, by recording any pharmacological and other interventions administered.
**Secondary objectives**
- To evaluate the Health-Related Patient-Reported Quality of Life (QoL) using the Kidney Disease Quality of Life Instrument KDQOL-SF v1.3 and the EQ-5D-5L index and VAS by assessing changes from baseline at 6 and 12 months.
- To assess possible changes in eGFR, if available and according to local practice, by calculating both CKD-EPI and MDRD eGFR values at baseline and at 6 and 12 months.
- To assess possible changes in UACR or microalbumin, if available and according to local practice, between baseline and at 6 and 12 months.

MDRD: modification of diet in renal disease; eGFR: estimated Glomerular Filtration Rate; CKD: chronic kidney disease; UACR: Urine Albumin Creatinine Ratio; CKD-EPI: CKD-EPI: Chronic Kidney Disease Epidemiology Collaboration; EQ-5D-5L = EuroQoL 5-dimension, 5-level; KDQOL-SF: Kidney Disease Quality of Life Short Form.

**Table 2:** Inclusion and exclusion criteria.

**Inclusion Criteria** Patients need to meet all of the following criteria to be included in the study:
- Provided signed informed consent form;
- Older than 18 years at the time of consent;
- CKD-EPI eGFR value between 15 and <60 mL/min/1.73 m^2^;
- Prior UACR or microalbumin measurement, regardless of value, within 6 months or at baseline according to clinical practice.
**Exclusion criteria** Patients need to meet none of the following criteria to be included in the study:
- Treatment for impaired renal function before recruitment (other than RAAS inhibitors, MRAs, immunosuppressants or immunomodulators);
- Management of CKD by a nephrologist prior to enrolment in the study;
- Patients already participating in another clinical trial;
- Pregnancy;
- Current treatment with SGLT2 inhibitor for any indication;
- Diabetes mellitus type 1;
- Renal transplantation prior to enrolment;
- Any condition outside the renal and cardiovascular study area with a life expectancy of <1 year based on investigator’s clinical judgement.

CKD: chronic kidney disease; CKD-EPI: Chronic Kidney Disease Epidemiology Collaboration; UACR: Urine Albumin Creatinine Ratio; MRAs: Mineralocorticoid Receptor Antagonists; SGLT2: Sodium-glucose cotransporter-2; RAAS: Renin–Angiotensin–Aldosterone System; eGFR: estimated Glomerular Filtration Rate.

**Table 3:** Patient demographic characteristics, comorbidities and hematological parameters at baseline.

	G3a *n* = 64 (37.9%)	G3b *n* = 67 (39.6%)	G4 *n* = 38 (22.5%)	Total *n* = 169 (100.0%)
Age (years)				
*n*	64	67	38	169
Mean (SD)	67.2 (12.1)	69.8 (12.7)	70.5 (14.1)	69.0 (12.8)
Gender, *n* (%)				
Male	52 (81.3)	51 (76.1)	18 (47.4)	121 (71.6)
Race, *n* (%)				
White/Caucasian	62 (96.9)	65 (97.0)	36 (94.7)	163 (96.5)
Asian	1 (1.6)	-	-	1 (0.6)
Black	-	-	1 (2.6)	1 (0.6)
Other	1 (1.6)	2 (3.0)	1 (2.6)	4 (2.4)
BMI ^[1]^ (kg/m^2^)				
*n*	64	67	38	169
Mean (SD)	28.5 (4.2)	28.1 (4.7)	28.2 (5.8)	28.2 (4.8)
Comorbidities ^[1],[2]^, *n* (%)				
Arterial Hypertension	45 (70.3)	46 (68.7)	30 (78.9)	121 (71.6)
Diabetes Mellitus Type 2	21 (32.8)	22 (32.8)	14 (36.8)	57 (33.7)
Coronary Artery Disease	11 (17.2)	15 (22.4)	5 (13.2)	31 (18.3)
Depression	2 (3.1)	2 (2.9)	1 (2.6)	5 (2.9)
Dyslipidemia	21 (32.8)	21 (31.3)	7 (18.4)	49 (28.9)
Hyperlipidemia	7 (10.9)	6 (8.9)	2 (5.3)	15 (8.9)
Hypothyroidism	4 (6.3)	7 (10.4)	6 (15.8)	17 (10.1)
Hyperuricemia	10 (15.6)	8 (11.9)	5 (13.2)	23 (13.6)
Atrial Fibrillation	2 (3.1)	7 (10.4)	5 (13.2)	14 (8.3)
Heart Failure	1 (1.6)	2 (2.9)	1 (2.6)	4 (2.4)
Osteoporosis	2 (3.1)	1 (1.5)	1 (2.6)	4 (2.4)
Missing	6 (9.4)	7 (10.4)	5 (13.2)	18 (10.7)
Hemoglobin (g/dL)				
*n*	59	51	30	140
Mean (SD)	13.1 (1.73)	12.7 (2.36)	11.5 (1.49)	12.6 (2.01)
Missing	5.0	16.0	8.0	29.0
Hematocrit (%)				
*n*	59	51	30	140
Mean (SD)	39.8 (4.79)	38.6 (6.71)	35.5 (4.45)	38.4 (5.70)
RBC (10^6^/μL)				
*n*	45	42	28	115
Mean (SD)	4.6 (0.67)	4.2 (0.70)	4.0 (0.45)	4.3 (0.68)
Missing	19.0	25.0	10.0	54.0
MCV (μm^3^)				
*n*	51	47	29	127
Mean (SD)	88.1 (6.27)	89.6 (7.32)	87.1 (6.39)	88.4 (6.72)
Missing	13.0	20.0	9.0	42.0
WBC (10^3^/μL)				
*n*	58	51	30	139
Mean (SD)	7.8 (7.90)	8.2 (2.70)	7.3 (1.85)	7.8 (5.45)
Missing	6.0	16.0	8.0	30.0
Platelets (10^3^/μL)				
*n*	59	50	30	139
Mean (SD)	248.4 (64.00)	249.4 (72.30)	247.0 (72.76)	248.4 (68.49)
Missing	5.0	17.0	8.0	30.0

CKD stages according to eGFR calculation, based on routine plasma creatinine measurement by hospital-based nephrologists. ^[1]^ BMI = (Weight (kg)/Height (cm)^2^) × 10,000. ^[2]^ Patients with more than one comorbidity are included in more than one row. SD = standard deviation; BMI= Body Mass Index; RBC = red blood cell count; WBC = white blood cell count; MCV = mean corpuscular volume.

**Table 4:** PRO questionnaires.

	G3a *n* = 64 (37.9%)	G3b *n* = 67 (39.6%)	G4 *n* = 38 (22.5%)	Total *n* = 169 (100.0%)
Health Questionnaire (EQ-5D-5L)				
Health Score (VAS Scale)	63 (98.4)	67 (100.0)	37 (97.4)	167 (98.8)
Mean (SD)	74.6 (18.9)	70.4 (18.8)	70.5 (15.8)	72.0 (18.3)
Kidney Disease and Quality of Life (KDQOL-SF)				
Health Rate (Scale)	62 (96.9)	67 (100.0)	37 (97.4)	166 (99.4)
Mean (SD)	7.0 (1.7)	6.7 (2.0)	6.5 (1.7)	6.7 (1.8)

CKD stages according to eGFR calculation, based on routine plasma creatinine measurement by hospital-based nephrologists. SD = standard deviation; VAS = Visual Analogue Scale; EQ-5D-5L = EuroQoL 5-dimension, 5-level.

**Table 5:** Treatment patterns.

	G3a *n* = 64 (37.9%)	G3b *n* = 67 (39.6%)	G4 *n* = 38 (22.5%)	Total *n* = 169 (100.0%)
Antibiotics ^[1], [3]^, *n* (%)	2 (3.1)	2 (2.9)	2 (5.3)	6 (3.6)
Antidiabetic ^[1], [3]^, *n* (%)	27 (42.2)	25 (37.3)	13 (34.2)	65 (38.5)
Metformin ^[2]^	15 (55.6)	4 (16.0)	2 (15.4)	21 (32.3)
DPP4 inhibitor ^[2]^	9 (33.3)	10 (40.0)	6 (46.2)	25 (38.5)
SGLT2 inhibitor ^[2], [4]^	5 (18.5)	8 (32.0)	1 (7.7)	14 (21.5)
GLP-1 analogue ^[2]^	6 (22.2)	4 (16.0)	-	10 (15.4)
Other ^[2]^	-	-	10 (76.9)	10 (15.4)
Antihypertensive ^[1], [3]^, *n* (%)	46 (71.9)	53 (79.1)	34 (89.5)	133 (78.7)
Adrenergic receptor antagonist ^[2]^	10 (21.7)	14 (26.4)	14 (41.2)	38 (28.6)
Calcium channel blocker ^[2]^	24 (52.2)	36 (67.9)	23 (67.6)	83 (62.4)
Angiotensin II receptor antagonist ^[2]^	36 (78.3)	22 (41.5)	6 (17.6)	64 (48.1)
Diuretic ^[2]^	16 (34.8)	9 (16.9)	11 (32.4)	36 (27.1)
ACE inhibitor ^[2]^	6 (13.0)	6 (11.3)	2 (5.9)	14 (10.5)
Other ^[2]^	10 (21.7)	11 (20.8)	10 (29.4)	31 (23.3)
Antiplatelet ^[1], [3]^, *n* (%)	17 (26.6)	19 (28.4)	5 (13.2)	41 (24.3)
Lipid-lowering agents ^[1], [3]^, *n* (%)	43 (67.2)	41 (61.2)	13 (34.2)	97 (57.4)
Immune suppressants/immunom odulators/NSAIDS ^[1], [3]^, *n* (%)	1 (1.6)	4 (5.9)	-	5 (2.9)
Other medications ^[1], [3]^, *n* (%)	14 (21.9)	23 (34.3)	11 (28.9)	48 (26.6)

CKD stages according to eGFR calculation, based on routine plasma creatinine measurement by hospital-based nephrologists. ^[1]^ Eligible patients were defined as all patients that were successfully assessed based on eligibility criteria. The number represents unique IDs of patients who received at least one of the listed treatments across all CKD stages. ^[2]^ Patients who received more than one treatment in this category are included in more than one row. ^[3]^ The total number of patients refers to unique patients in each treatment category. ^[4]^ SGLT2 inhibitors received as treatment for one or more of their indications. T2D, heart failure or CKD abbreviations: SGLT2: Sodium-glucose cotransporter-2; RAAS: Renin-Angiotensin-Aldosterone System; eGFR: estimated Glomerular Filtration Rate; DPP4: Dipeptidyl peptidase-4; GLP-1: glucagon-like peptide-1; ACE: angiotensin-converting enzyme; NSAIDS: nonsteroidal anti-inflammatory drugs.

## Data Availability

The datasets used and analyzed during the current study are available from the corresponding author on reasonable request.
